# Relationship between Duration of Pulp Exposure and Success Rate of Apexogenesis

**Published:** 2007-01-20

**Authors:** Saeed Moradi, Neda Naghavi, Ehsan Roohani, Nooshin Mohtasham

**Affiliations:** 1 *Department of Endodontics, Dental Research Center, Dental School, Mashad University of Medical Sciences, Mashad, Iran*; 2 *Department of Endodontics, Dental School, Mashad University of Medical Sciences, Mashad, Iran*; 3 *Department of Restorative Dentistry, Dental School, Mashad University of Medical Sciences, Mashad, Iran*; 4 *Department of Oral Pathology, Dental School, Mashad University of Medical Sciences, Mashad, Iran*

**Keywords:** Apexogenesis, Calcium Hydroxide, MTA

## Abstract

**INTRODUCTION:** Apexogenesis is a way to save vitality of open apex damaged teeth with mild or moderate pulp involvement. Such teeth are not repaired through normal and usual treatments. This treatment provides usual and physiological conditions for root to develop in normal length. The aim of this study was to determine the success rate of apexogenesis according to the duration of pulp exposure.

**MATERIALS AND METHODS:** In this animal study, mineral trioxide aggregate (MTA) and calcium hydroxide (CH) were used. The examined teeth were canines of cats with open apices. The treatment was accomplished in three periods of 1, 3, and 6 weeks after pulpal exposure. Four months later, the results were evaluated histologically and radiographically.

**RESULTS:** The results showed no significant difference between the success rate of MTA and CH. Besides, after 6 weeks of pulpal exposure the treatment was successful. Root development and apical closure was detected in approximately 42% of teeth, while 33% of samples had a healthy Hertwig’s sheath.

**CONCLUSION:** The findings of this study suggested that the conservative treatment in traumatized teeth after 1.5 month of pulpal exposure could be successful.

## INTRODUCTION

The Treatment of open apex teeth is one of the most difficult and important subjects in endodontics treatments. An important factor in such treatments is the rate and severity of pulpal inflammation. Numerous studies have evaluated the progress of inflammation in pulp and the periapical region of closed apex teeth. Their results have shown that one week after pulp exposure, the adjacent area to the exposure point becomes necrotic and congestive and after four weeks, almost all parts of the pulp become necrotic (1-7). A healthy pulp is essential for the proper development of the root (8).

It seems that the pulp of an open apex tooth is potentially more resistant against various irritations. Thus, this may cause its longer survival after a hard trauma rather than the pulp of a closed apex tooth (9). The condition of Hertwigʼs epithelial root sheath is another important factor that affects the treatment of an open apex tooth. In evaluation of periapical condition and Hertwigʼs sheath, it has been defined that one month after pulp involvement, Hertwigʼs sheath remains healthy (10).

Root growth was only possible when the Hertwigʼs epithelial root sheath has retained its specialized function (11).

Various materials have been used in pulp capping and pulpotomy procedures (12-14). calcium hydroxide (CH) is the most frequently used material since 1920.

Nowaday, newer materials are preferred for vital pulp therapy, such as MTA (15). They stimulate significantly greater hard-tissue formation in the periradicular tissues and result in less inflammation in comparison with the use of CH (16).

Maintaining the vitality of immature teeth until their full-root development is so important. Loss of vitality of these teeth before root completion leaves poor crown/root ratio, weak roots more prone to fracture, and teeth more susceptible to periodontal breakdown.

The findings of this study may help us to plan a more conservative treatment for such teeth which were referred to us, at different periods after trauma.

Thus the aim of this study was to evaluate the MTA and CH used as pulpotomy agent in open apex tooth of cats at different times after pulp exposure.

## MATERIALS AND METHODS

Eleven one-year-old cats with permanent open apex canine teeth have been selected for this study. The treatment was done on their canine teeth. They were divided into three groups: a one-week group with 4 cats, a three-week group with 4 cats and a six-week group with 3 cats.

The cats were given general anesthesia with a mixture of 0.8cc Ketamin hydrochloride 50mg/ml and 0.2cc of 2% Rompun (a relaxant). After 3-5 minutes, intraoral anesthesia was achieved by injection of 1.8 ml of 2% Lidocaine containing 1:100,000 epinephrine. A radiographic image was taken to confirm the opening of the apex of the teeth. Using a high speed fissure bur number 8 (Tizkavan, Tehran, Iran) and copious water spray, the crowns were ground at the incisal part until a very small exposure about 0.5mm in diameter was achieved. These teeth were exposed according to the respected group for 1, 3, or 6 weeks.

Subsequent treatment was performed as follows: The cats were anesthetized again. The cavity access was prepared. Then the pulpotomy was done until vital pulp tissue and normal bleeding was detected. The necrotic and inflamed pulp tissue was excavated completely. The apexogenesis materials (MTA and CH) were put on the healthy pulp as follows: CH on the right upper and lower teeth, MTA on the left upper and lower teeth. A double-seal method with glass ionomer and amalgam was used for coronal seal. In each group one tooth was left intact as control. Radiographic records have been taken and animals were followed. Four month later, after general anesthesia, another radiograph was prepared for each tooth. Then, the vital perfusion was processed. Finally, the histological slides were prepared and pulpal inflammation, periapical inflammation, Hertwig’s sheath health status, and root development were evaluated.

For evaluation of the inflammation severity, an area of 100 square micrometers was selected from the most inflammatory region in the periapical zone, and plasma cells, lymphocytes, macrophages and polymorphonuclears were counted with x400 magnification. Considering the number of cells, four categories were developed:

1- Without inflammation: 0-1 cell in 100 µm^2^

2- Slight inflammation: 2-5 cell in 100µm^2^

3- Moderate inflammation: 6-9 cell in 100 µm^2^

4- Severe inflammation: over 9 cells in 100 µm^2^

The type of inflammation was determined according to the type of infiltrative cells as:

a- Chronic inflammation: plasma cells, lymphocytes, macrophages

b- Acute inflammation: polymorphonuclears

*Chi-Square* test was used in all statistical analysis.

## Results

**Figure 1:**
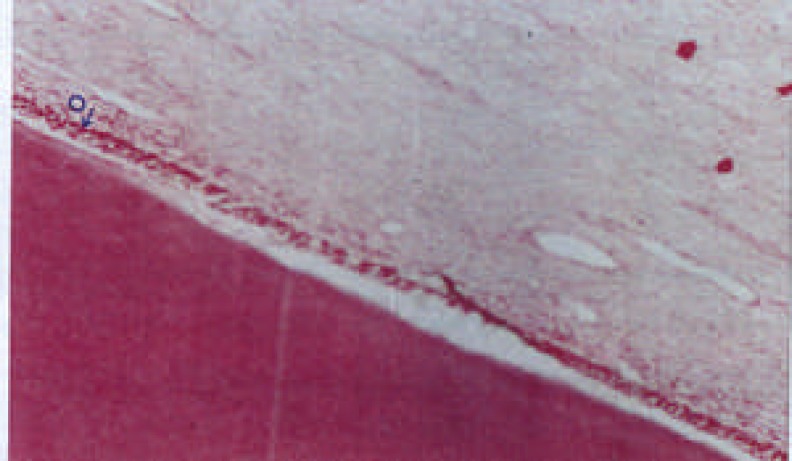
Intact sample, normal pulp without inflammation D: dentin, O: ododontoblastic layer, P: Pulp (×100)

In all control teeth the pulp was normal and without any inflammation. The odontoblastic layer and the development of the root were normal and the epithelial root sheath had normal patterns (Figure 1).The results of histo-pathological evaluation are shown in table 1.

**Figure 2:**
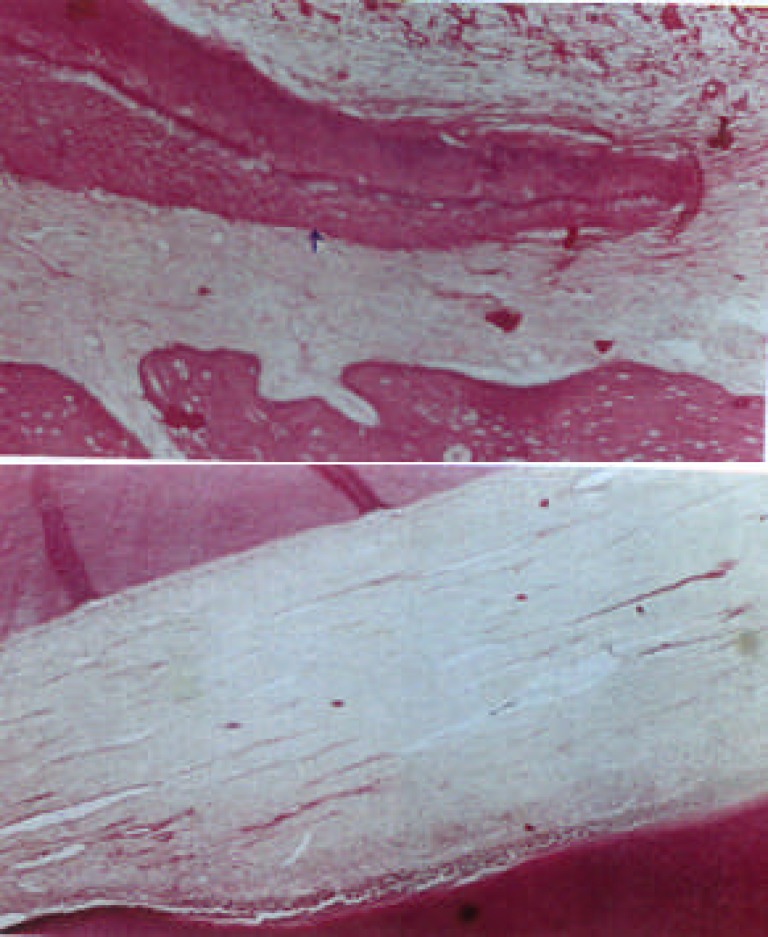
One week period samples treated with MTA A: Healthy Hertwig sheath (×400) B: Healthy pulp (×400)

**Figure 3:**
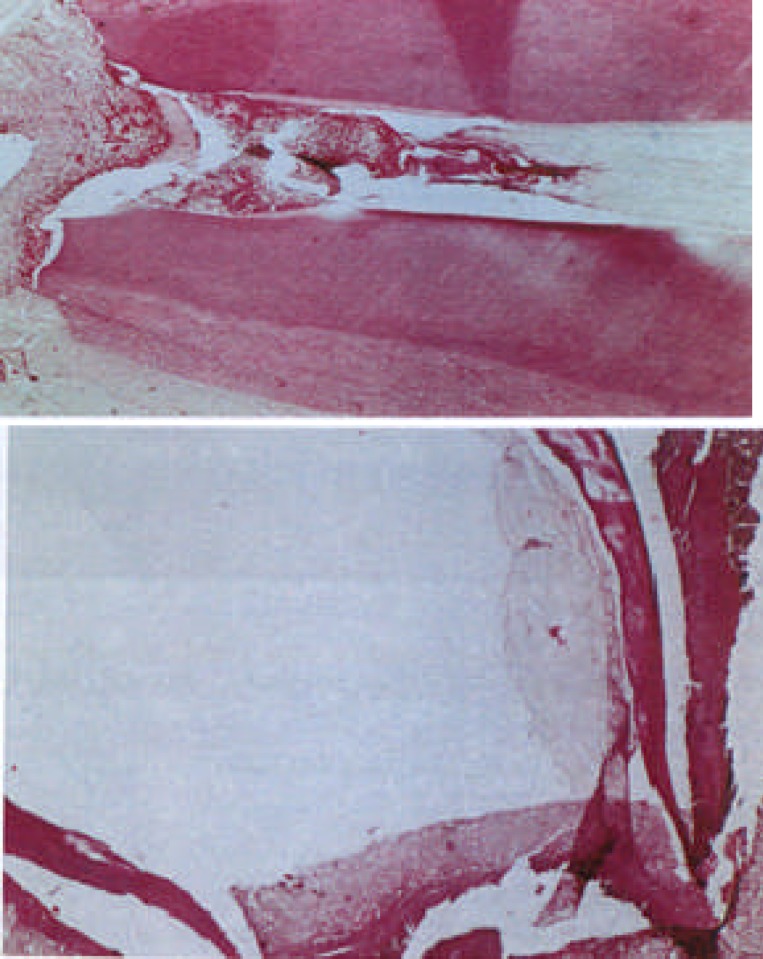
Three week period samples treated with MTA A: Development of root with thick dentinal wall and close apex (×100) B: Incomplete of root with open apex and thin dentinal wall (×100)

**Figure 4:**
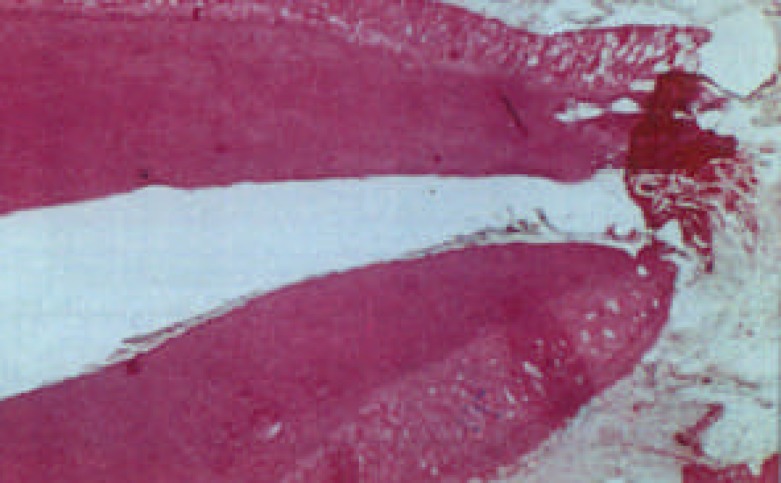
Three week period sample treated with Ca(OH)2 Pulp completely destroyed but apex was closed (×400)

**Figure 5:**
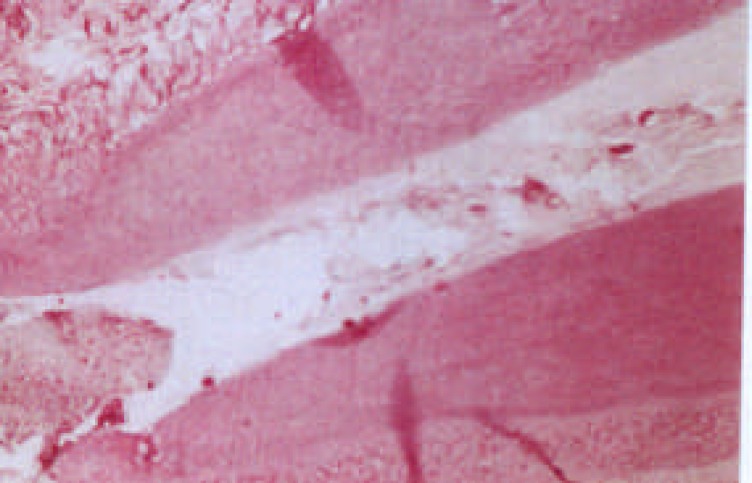
Six week period sample treated with MTA Apical region P: pulp, CA: closed apex (×400)

**Figure 6:**
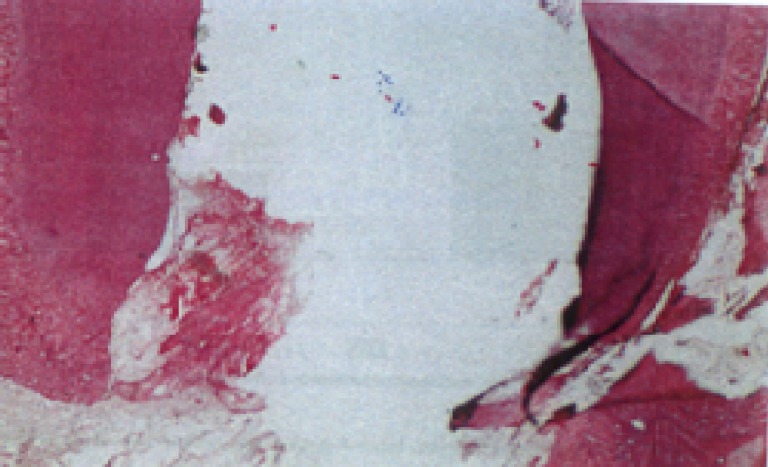
Six week period samples treated with Ca(OH)2. Begging of apex closure and thicken the dentinal wall (×400)

Comparison of the two materials at the same time: In one-week group there was a significant difference between two materials only in the conditions of Hertwig's sheath (P<0.05). In MTA-treated group, the Hertwig's layer condition was better (Figure 2). In three and six-week groups there was no significant difference between two materials in any of four factors (P>0.05) (Figure 3, Figure 4, Figure 5, Figure 6).

Comparison of the two materials regardless of time: There was no significant difference between two materials in 4 studying factors (p>0.05).

Comparison of three periods of time with the same material: In Both groups there was no significant difference in studying factors according to three time periods (p>0.05).

**Table 1:** The results of histopathological evaluation in three periods of study

		**One- week group**		**Three-week group**		**Six-week group**	
		**MTA**	**Ca(OH)2**	**MTA**	**Ca(OH)2**	**MTA**	**Ca(OH)2**
**Inflammation of pulp**	*Necrosis*	80%	14.2%	75%	62.5%	83.3%	83.3%
	*Severe*	20%	57.1%	25%	25%	16.7%	-
	*Moderate*	-	-	-	-	-	-
	*Mild*	-	14.2%	-	-	-	-
	*Healthy*	-	14.2%	-	12.5%	-	16.7%
**Inflammation of periapical**	*Severe*	40%	28.4%	75%	37.5%	100%	50%
	*Moderate*	40%	42.8%	12.5%	50%	-	33.3%
	*Mild*	-	14.2%	12.5%	-	-	-
	*Healthy*	20%	14.2%	-	12.5%	-	16.7%
**Condition of Hertwig´s sheath**	*Destroyed*	20%	71.5%	37.5%	50%	-	33.3%
	*Being destroyed*	-	28.5%	37.5%	25%	66.6%	66.7%
	*Healthy*	80%	-	25%	25%	33.4%	-
**Closure of apex**	*Open*	-	14.5%	12.5%	37.5%	50%	66.6%
	*Being closed*	-	-	-	-	-	33.4%
	*Closed*	100%	85.5%	87.5%	62.5%	50%	-

Comparison of the results in each time period regardless of the type of material: There was a significant difference only in the rate of apex closure in three time intervals (p<0.05). The results in one-week group were better than those of three and six-week groups, and in three weeks group the results were better than that of six week group.

Comparison of the results of upper and lower jaws with a constant material: Neither MTA nor Ca (OH)2 showed significant difference in studying factors between upper and lower jaws (p>0.05).

## DISCUSSION

It is known that Hertwigʼs root sheath can organize the apical cells and causes continued formation of the root even after the pulp necrosis. This demonstrated that the root sheath is not destroyed (17).

Cvek stated that the root sheath is usually sensitive to trauma; however, in some circum-stances it may resist against damages from trauma and infection (18).

In a study on open apex canine teeth of cats with MTA, CH and formocresol, accomplished by Ghodduci* et al.*, there was a significant difference between the rate of inflammation and root development in MTA and CH treated groups. In all of the treated cases with MTA, the radicular pulp of the root was normal and in 88.9% of them the root had been developed completely, but in CH treated group, only 75% of the teeth had a vital pulp (19). Time interval between the pulp exposure and apexogenesis in our study may be the reason of differences between our results with theirs. Our findings were more dependent on passed exposure time.

In the study of Thomas *et al.* (20) on 12 incisor teeth of monkey and the study of Abedi *et al.* (16) on dog's canine teeth, there was less pulpal inflammation in MTA treated group. Time factor could be attributed in this study too.

In this study, treatment of some teeth in the 6-weeks group was successful and the development of root was going on. This considerable finding can affect the treatment plan of the open apex tooth with no positive response to the vital tests at first observation.

In spite of trauma and infection, the Hertwigʼs root sheath remained viable and continued to map out the apical segment resulting in root end development (21).

Andreasen *et al.* suggested that by complete removing of microorganisms and applying a material_ which is not irritant for the periapical tissue_ in the root canal, Hertwigʼs sheath may continue root-end completion in an apparently normal manner (22).

We can manage these teeth with a conservative treatment and saving Hertwig’s sheath in order to complete physiological root development.

## CONCLUSION

According the results of this in vitro study, the conservative treatment of pulpally exposed traumatized teeth after 6 weeks can successful.
